# Unusual association of ST-T abnormalities, myocarditis and cardiomyopathy with H_1_N_1 _influenza in pregnancy: two case reports and review of the literature

**DOI:** 10.1186/1752-1947-5-314

**Published:** 2011-07-14

**Authors:** Karen Chan, David Meek, Indranil Chakravorty

**Affiliations:** 1 Department of Respiratory Medicine, Lister Hospital, Corey's Mill Lane, Stevenage, UK; 2 School of Postgraduate Medicine, University of Hertfordshire, Health Research Building, College Lane Campus, Hatfield, UK

## Abstract

**Introduction:**

Myocarditis is rarely reported as an extra-pulmonary manifestation of influenza while pregnancy is a rare cause of cardiomyopathy. Pregnancy was identified as a major risk factor for increased mortality and morbidity due to H_1_N_1 _influenza in the pandemic of 2009 to 2010. However, to the best of our knowledge there are no previous reports in the literature linking H_1_N_1 _with myocarditis in pregnancy.

**Case presentation:**

We report the cases of two pregnant Caucasian women (aged 29 and 30), with no pre-existing illness, presenting with respiratory manifestations of H_1_N_1 _influenza virus infection in their third trimester. Both women developed evidence of myocarditis. One woman developed acute respiratory distress syndrome, almost reaching the point of requiring extra-corporeal membrane oxygenation, and subsequently developed persistent cardiomyopathy; the other recovered without any long-term consequence.

**Conclusions:**

While it is not possible to ascertain retrospectively if myocarditis was caused by either infection with H_1_N_1 _virus or as a result of pregnancy (in the absence of endomyocardial biopsies), the significant association with myocardial involvement in both women demonstrates the increased risk of exposure to H_1_N_1 _influenza virus in pregnant women. This highlights the need for health care providers to increase awareness amongst caregivers to target this 'at risk' group aggressively with vaccination and prompt treatment.

## Introduction

Many previous studies have explored the link between influenza and myocarditis. Influenza virus (along with Coxsackie B, adenovirus, echovirus and cytomegalovirus) has long been a recognized cause of myocarditis. Myocarditis can manifest in varying severity, ranging from a mild rise in myocardial enzymes to presenting with profound cardiogenic shock. Previous studies investigating influenza pandemics have confirmed multiple organ involvement on autopsy, including myocarditis and pericarditis. A pandemic caused by the H_1_N_1 _type influenza virus has been a topic of great interest of late. Treatment with osteltamivir shortened the period of infection. To date, only one study has explored the association of myocarditis in H_1_N_1 _infection in children. This highlighted that there should be a high index of suspicion for myocarditis in children with H_1_N_1 _influenza A infection. It emphasized the importance of early detection and aggressive management. Timely intervention with circulatory support was said to perhaps decrease morbidity and mortality, with potential for a favorable cardiac prognosis [1].

## Case presentations

Two pregnant women were admitted to our hospital in 2009 with a history of an acute viral-like illness.

**Figure 1:**
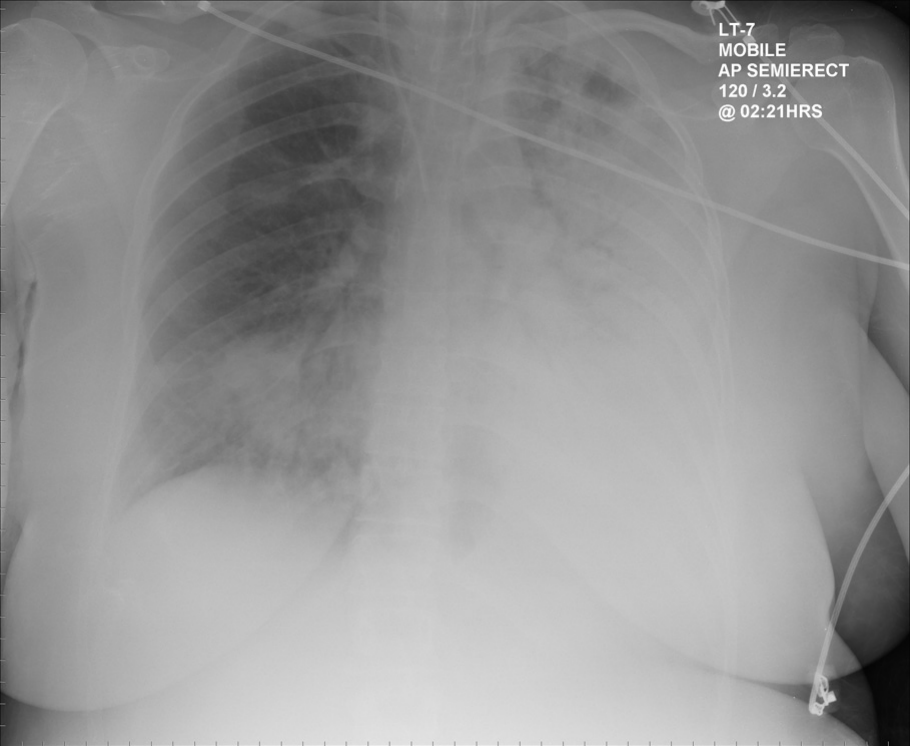
**Chest radiograph of our first patient demonstrating an infective infiltrate**.

Our first patient was a 30-year-old Caucasian woman who presented at 28 weeks' gestation with a four-day history of pyrexia (spiking at 40°C) and shortness of breath. Aside from childhood bronchitis, there was no other relevant medical or surgical history. Examination revealed reduced breath sounds and bronchial breathing in the left base. Her C reactive protein (CRP) level was raised, with a mildly raised white cell count. A chest radiograph (Figure 1) showed consolidation and collapse of the left lower lobe. Arterial blood gas levels taken at the time were consistent with a severe type 1 respiratory failure. As a result of her severe hypoxia, she was electively intubated and ventilated. In view of her deteriorating status, her baby was delivered by emergency Caesarean section with no immediate post-operative complications. From admission, she was treated with antimicrobials and osteltamivir. She was also swabbed and subsequently confirmed as being H_1_N_1 _positive.

Post-operatively whilst in intensive care, she proved difficult to oxygenate and ventilate. Therefore, she was transferred to Glenfield Hospital (Leicester, UK) for consideration of extracorporeal membrane oxygenation (ECMO). However, she did not need ECMO and improved on conventional mechanical ventilation.

Our patient was transferred back to our hospital for further convalescence. An electrocardiogram was performed, which revealed sinusoidal and anteroinferior ST elevation. Her troponin levels returned negative. She was referred for an urgent echocardiogram, which demonstrated preserved overall biventricular systodiastolic function. She made a good recovery from this episode and was seen as an out-patient, where she was found to have persisting symptoms of myocardial dysfunction; namely Medical Research Council (MRC) class II to III dyspnea, chest pain and palpitations. She had a repeat echocardiogram, which confirmed preserved left and right ventricular function, and is awaiting further cardiac investigations.

**Figure 2:**
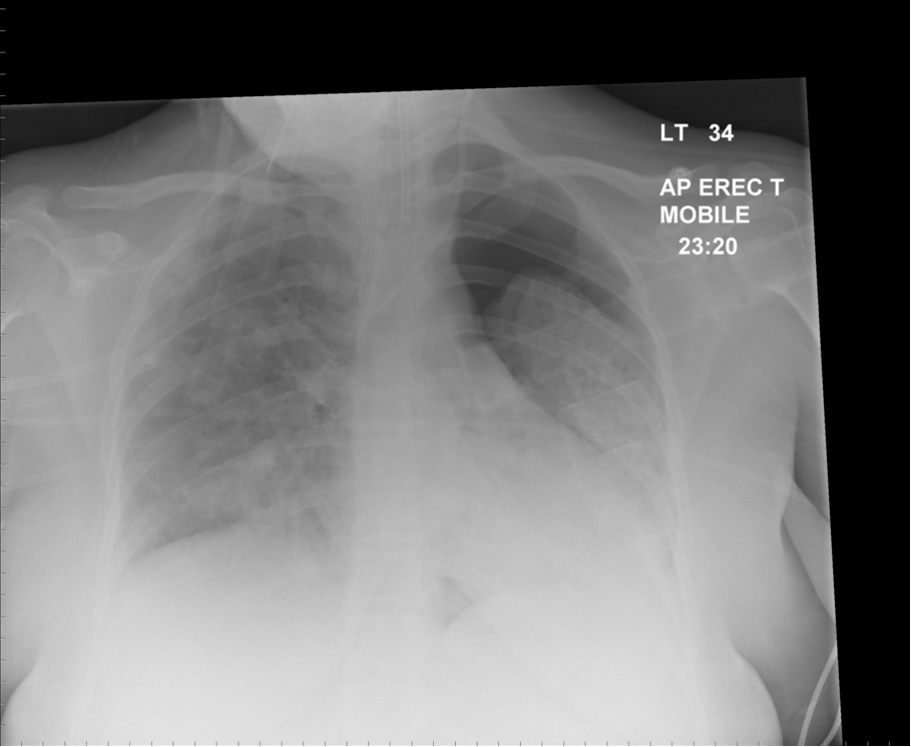
**Chest radiograph of our first patient demonstrating a pneumothorax**.

**Figure 3:**
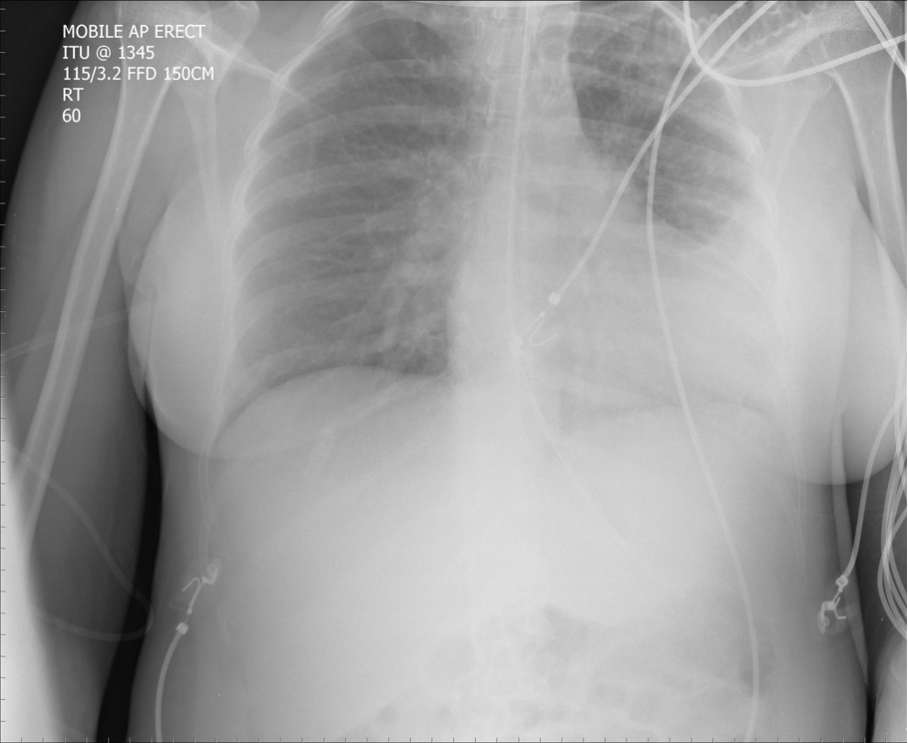
**Chest radiograph demonstrating infective infiltrate/consolidation**.

Our second patient was a 29-year-old Caucasian woman who was admitted by our Obstetric team with a five-day history of pyrexia and vomiting. On admission she was 37 weeks' pregnant. She had no medical or surgical history of note. On examination, she had bronchial breathing in the entire left lung and the right mid and lower zones. Her CRP level was raised with a moderately raised white cell count. A chest radiograph at this point revealed dense multi-lobular shadowing and consolidation (Figures 2 and 3) and she was started on intravenous antibiotics and zanamivir. Osteltamivir was added at a later date. As in our first patient, she continued to deteriorate and developed severe type 1 respiratory failure requiring her transfer to our intensive care unit and invasive ventilation. In light of her deteriorating clinical condition, her baby was delivered by emergency caesarean section. She suffered no immediate post-operative complications and her child was healthy.

Whilst in the intensive care unit, our patient also suffered from a persistent left sided pneumothorax (Figure 3) requiring an intercostal chest drain. Furthermore, she was noted to have T wave inversion in her anterior and lateral leads. A troponin test was negative. Her creatinine kinase levels were also within the normal range. She underwent an echocardiogram, which showed global hypokinesia and moderate to severely impaired left ventricular systolic function. Subsequent repeat echocardiograms confirmed persistent left ventricular (LV) systolic dysfunction. As a result, she was commenced on treatment with an angiotensin converting enzyme inhibitor (ACE-I). A repeat echocardiogram still showed moderately impaired LV function (ejection fraction estimated at 35%). Despite this, our patient made a good recovery and was discharged from hospital.

She was followed up as an out-patient by both the Respiratory and Cardiology departments and was clinically making good progress. Her repeat echocardiogram revealed continuing moderate to severe left ventricular function.

## Discussion

Uncomplicated human influenza virus infection causes transient tracheobronchitis, corresponding with predominant virus attachment to tracheal and bronchial epithelial cells. The main complication is extension of viral infection to the alveoli, often with secondary bacterial infection, resulting in severe pneumonia and often extending to adult respiratory distress syndrome (ARDS). Complications in extra-respiratory tissues such as encephalopathy, myocarditis, and myopathy occur occasionally [2,3]. The association of a severe influenza-like illness followed by the development of myocardial dysfunction or cardiomyopathy has been described in 20% of patients in epidemiological studies [4,5] and also recognized via a rise in antibody titers in association with pregnancy [6].

In patients with suspected viral myocarditis, echocardiography and electrocardiographic abnormalities are usually seen in 29% to 33% [7]. Physiological changes associated with pregnancy is recognized as one of the factors reducing the efficiency of T helper cells thus increasing the risk of mortality from influenza [8]. Murine studies indicate that the acute cardiac injury is related to cytotoxic immunologic interactions, virus-induced cytolysis and, to ischemia due to intra-capillary thrombosis [9], while myocarditis is caused frequently by viral infections of the myocardium [10].

In the past, enteroviruses (EV) were considered the most common cause of myocarditis in all age groups. Other viruses that cause myocarditis are adenovirus, influenza, parvovirus B19, members of the Herpesviridae family, cytomegalovirus (CMV), and human herpesvirus 6 (HHV-6) have all been associated occasionally with myocarditis [11]. Viral genomes are frequently detected by polymerase chain reaction enhancement in endomyocardial biopsies of patients with systolic left ventricular dysfunction and this may play a role in the pathogenesis of cardiomyopathy far more frequently [12,13].

Acute H_1_N_1 _infections in pregnancy have been reported in the current pandemic leading to severe morbidity, as seen in our two patients, and mortality [14,15]. The fact that this influenza A (H_1_N_1_) can develop in healthy patients and evolve in few hours to a severe ARDS with a refractory hypoxemia needing recourse to ECMO in 5% to 20% of patients is novel [16,17]. The first publications of patients admitted to intensive care units for severe influenza A (H_1_N_1_) often associated to an ARDS reported a mortality rate from 15% to 40% [18].

In California, data were reported for 94 pregnant women, eight post-partum women, and 137 non-pregnant women of reproductive age who were hospitalized with 2009 H_1_N_1 _influenza. Most patients who were pregnant (95%) were in the second or third trimester, and approximately one-third (34%) had established risk factors for complications from influenza other than pregnancy. As compared with early antiviral treatment (administered before or at two days after symptom onset) in pregnant women, later treatment was associated with admission to an intensive care unit or death (relative risk, 4.3). In all, 22% required intensive care, and 8% died [19]. The estimated rate of admission for pandemic H_1_N_1 _influenza virus infection in pregnant women during the first month of the outbreak was higher than it was in the general population. Between 15 April and 16 June 2009, six deaths in pregnant women were reported to the Centre for Disease Control, USA; all were in women who had developed pneumonia and subsequent acute respiratory distress syndrome requiring mechanical ventilation [20].

Although influenza virus is a rare but recognized cause of myocarditis and pregnancy is a known risk factor for the development of peri-partum cardiomyopathy, the association of H_1_N_1-_associated severe viral pneumonia combined with features of troponin negative myocarditis and cardiomyopathy in our two consecutive patients raises the novel and hitherto unreported association between H_1_N_1 _infection and myocardial involvement which increases the risk significantly for pregnant women. The absence of an acute rise in cardiac enzymes and the low sensitivity of transthoracic echocardiography in recognizing myocarditis may be detrimental to early recognition and institution of appropriate treatment as may be seen in up to two out of three patients.

Obstetric providers need to be prepared to provide the care necessary to address the increased morbidity, mortality, and pregnancy-related complications (including spontaneous miscarriage and pre-term birth) faced by pregnant women during an influenza pandemic [21]. Many obstetric health care workers often lack knowledge regarding the safety and importance of influenza vaccination during pregnancy. Misinformed or inadequately informed health care workers may represent a barrier to influenza vaccine coverage of pregnant women. This lack of knowledge among the health care workforce takes on added importance in the setting of the H_1_N_1 _2009 swine-origin influenza pandemic [22]. Inactivated influenza vaccine can be safely and effectively administered during any trimester of pregnancy. No study to date has demonstrated an increased risk of either maternal complications or adverse fetal outcomes associated with inactivated influenza vaccination. Moreover, no scientific evidence exists that thimerosal-containing vaccines are a cause of adverse events among children born to women who received influenza vaccine during pregnancy [23]. Maternal influenza immunization is a highly cost-effective intervention at disease rates and severity that correspond to both seasonal influenza epidemics and occasional pandemics. These findings justify ongoing efforts to optimize influenza vaccination during pregnancy from an economic perspective [24].

## Conclusions

These two cases of H1N1 infection in relatively normal pregnant women illustrate the increased risk of life-threatening complications (including myocarditis and cardiomyopathy) in this group and the multi-system involvement seen. Thus, increased awareness amongst patients and health care professionals and a higher uptake of prevention strategies may result in improved survival in future epidemics.

## Consent

Written informed consent was obtained from both the patients for publication of this case report and any accompanying images. A copy of the written consent is available for review by the Editor-in-Chief of this journal.

## Competing interests

The authors declare that they have no competing interests.

## Authors' contributions

KC drafted the manuscript and researched the case. DM supervised the drafting of the report, revised the draft copy of the manuscript and reviewed the medical literature surrounding this case. IC supervised, contributed to the literature review, revised the report and gave final approval for the manuscript to be submitted.

All authors have read and approved the final manuscript.
